# An Effector Peptide Family Required for *Drosophila* Toll-Mediated Immunity

**DOI:** 10.1371/journal.ppat.1004876

**Published:** 2015-04-27

**Authors:** Alexa W. Clemmons, Scott A. Lindsay, Steven A. Wasserman

**Affiliations:** Section of Cell and Developmental Biology, Division of Biological Sciences, University of California, San Diego, La Jolla, California, United States of America; University of Massachusetts, Worcester, UNITED STATES

## Abstract

In *Drosophila melanogaster*, recognition of an invading pathogen activates the Toll or Imd signaling pathway, triggering robust upregulation of innate immune effectors. Although the mechanisms of pathogen recognition and signaling are now well understood, the functions of the immune-induced transcriptome and proteome remain much less well characterized. Through bioinformatic analysis of effector gene sequences, we have defined a family of twelve genes – the *Bomanins* (*Boms*) – that are specifically induced by Toll and that encode small, secreted peptides of unknown biochemical activity. Using targeted genome engineering, we have deleted ten of the twelve *Bom* genes. Remarkably, inactivating these ten genes decreases survival upon microbial infection to the same extent, and with the same specificity, as does eliminating Toll pathway function. Toll signaling, however, appears unaffected. Assaying bacterial load post-infection in wild-type and mutant flies, we provide evidence that the *Boms* are required for resistance to, rather than tolerance of, infection. In addition, by generating and assaying a deletion of a smaller subset of the *Bom* genes, we find that there is overlap in *Bom* activity toward particular pathogens. Together, these studies deepen our understanding of Toll-mediated immunity and provide a new *in vivo* model for exploration of the innate immune effector repertoire.

## Author Summary

Dedicated defense systems in the bodies of humans and other animals protect against dangerous microbes, such as bacteria and fungi. We study these processes in the fruit fly *Drosophila*, which can be readily grown and manipulated in the laboratory. In this animal, as in humans, protective activities are triggered when fragments of bacteria or fungi activate a system for defense gene regulation known as the Toll signaling pathway. The result is the large-scale production of defense molecules and, in many cases, clearance of the infection and survival of the animal. Although the systems for recognizing and initiating responses are well described, the role of many defense molecules is not understood. We have identified a group of closely related defense molecules in flies and used state-of-the-art genomic engineering to simultaneously eliminate most of the genes in the group. By comparing the effect of fungal or bacterial infection on the genetically altered flies and normal siblings, we find that this group of defense molecules is essential for disease resistance.

## Introduction

Constant interaction with microbes is a fact of life, and sometimes death, for animals. Many microbes are neutral or beneficial to the host’s health. Some, however, are pathogenic and threaten the host’s viability. In vertebrates and invertebrates alike, immune responses are initiated by recognition of pathogen associated molecular patterns (PAMPs) following invasion of host tissues [1, 2]. This recognition of conserved microbial products triggers innate immune signaling pathways that are closely related in species as divergent as flies and humans [3–5]. In each case, pathway activation initiates a transcriptional program encoding an array of effector peptides and proteins.

In the fruit fly *Drosophila melanogaster*, Toll and Imd proteins define the two major immune signaling pathways [6–11]. Fragments of fungal cell walls and bacterial peptidoglycan serve as PAMPs for these pathways. Toll signaling is triggered by the β-1,3-glucans of fungal cell walls or by Lys-type peptidoglycan [12–16]. In contrast, the Imd pathway is activated by DAP-type peptidoglycan [17–21]. Upon activation, Toll and Imd direct expression of distinct but overlapping effector gene repertoires. These effector genes bring about the humoral immune response via factors, including antimicrobial peptides (AMPs), that circulate throughout the fly hemolymph. Effector genes also support other immune processes by, for example, upregulating genes promoting melanization and wound healing [22].

The *Drosophila* immune effector repertoire has been characterized by microarray, RNA-seq, and mass spectrometry experiments [23–27]. The most highly upregulated genes include most known AMPs, but also many as yet uncharacterized effector peptides. For both the characterized and novel effectors, delineation of *in vivo* requirements based on loss-of-function phenotypes is largely lacking.

Here, we describe the application of recent advances in genome engineering technology to the genetic dissection of innate immune effector function. Generating a designer deletion of multiple members of an effector gene family, we demonstrate an essential role for these genes in Toll-mediated defense against microbial pathogens.

## Results

### The *Bom* genes encode a family of short, secreted, Toll-regulated peptides

**Fig 1:**
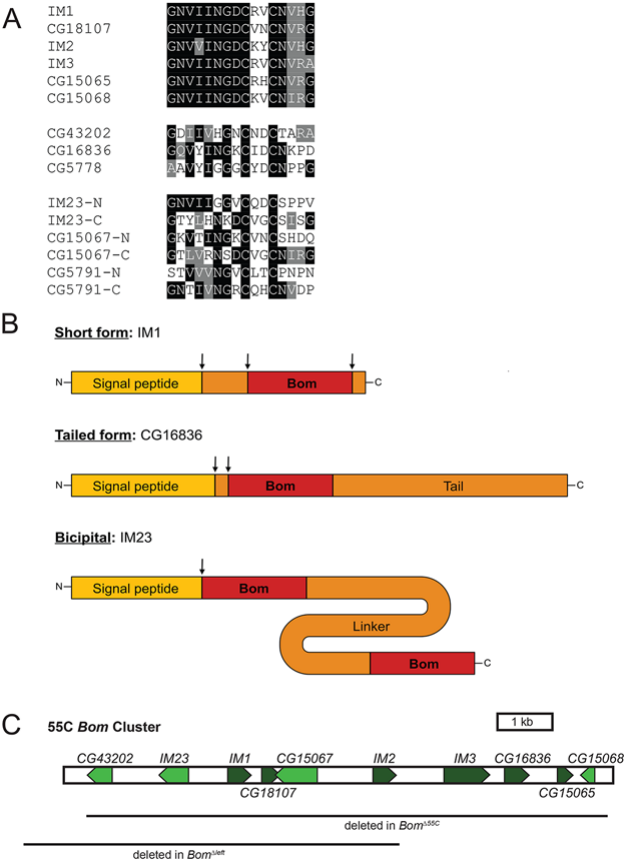
*Bom* genes share a conserved 16-aa motif. (A) Alignment of Bom motifs. Top. Mature Bom peptide sequences of the short-form Boms. Middle. Bom peptide motifs of the tailed Boms. Bottom. Bom peptide motifs from the N- and C-terminal ends of the three bicipital Boms. Shading indicates sequence identity (black) or similarity (gray). (B) Schematic of the three Bom peptide forms. ‘Bom’ represents the conserved 16-aa motif depicted in Fig 1A. Drawings are to scale and arrows indicate sites of cleavage. (C) Schematic of 55C *Bom* gene cluster on chromosome 2R. Lines beneath schematic demarcate areas deleted in *Bom* ^*Δ55C*^ and *Bom* ^*Δleft*^ chromosomes. The proximal end of the gene cluster is shown to the left.

Carrying out sequence comparisons among *Drosophila melanogaster* loci induced by the Toll pathway [24], we identified a family of twelve genes encoding secreted peptides lacking similarity to known AMPs. Each of the twelve peptides contains one or two copies of a 16 amino acid-long motif that includes a CXXC bend surrounded by a region of high sequence conservation (Fig 1A). All orthologs identified to date are from members of the *Drosophila* genus. We propose naming this family of genes the *Bomanins* (*Boms*), after Hans Boman, who carried out pioneering work in peptide-mediated innate immunity [28–31].

Several Bom peptides belong to the set of Immune-induced Molecules (IMs) first identified in mass spectrometry studies carried out by Bulet, Hoffmann, and colleagues [23, 25]. Combining those findings with detailed sequence comparisons reveals post-translational processing events: signal peptide cleavage and, often, removal of additional residues at the amino-terminal end as well as carboxyl-terminal amidation (Fig 1B).

The Bom peptides fall into three distinct groups (Fig 1B and S1 Table). For six of the twelve, the mature peptide is just 16 or 17 amino acids long. The sequences of these six *short-form* peptides are highly similar and correspond to the conserved, CXXC-containing region that we have defined as the Bom motif (see Fig 1A and 1B). Three other Bom peptides have a *tailed form*—a Bom motif followed by a C-terminal extension or tail, 15 to 82 amino acids in length. The remaining three peptides have a Bom motif at each end, connected by a linker region of 43 to 103 amino acids. We refer to these peptides as two-headed or *bicipital*. Sequence identity and similarity within the Bom motif is reduced, but still significant, in the tailed and bicipital forms (see Fig 1A). In contrast, the tail and linker regions in these two classes are rich in homopolymeric stretches and contain no appreciable sequence conservation either with each other or with other proteins in available databases.

Published microarray, RNA-seq, and mass spectrometry experiments document robust expression of the *Bom* transcripts and peptides after bacterial or fungal infection [22–27]. Indeed, induced expression of many *Boms* is at levels equal to or greater than those of AMP loci. Furthermore, Bom peptides, like AMPs, are abundant in the hemolymph of infected flies [23, 32].

Ten of the twelve *D*. *melanogaster Bom* genes are clustered on chromosome 2 at cytogenetic position 55C (henceforth 55C *Bom* cluster, Fig 1C). The two remaining *Bom* genes, *CG5791* and *CG5778*, reside in a mini-cluster on chromosome 3 and encode a bicipital and a tailed Bom peptide, respectively. Because the predicted mature Bom peptides are highly similar and hence potentially overlapping in function, we began our investigation of the *Boms* by precisely deleting the ten genes of the 55C *Bom* cluster using a TALEN-based approach. The deletion, henceforth *Bom* ^*Δ55C*^, is 9 kb long and removes no annotated loci other than the *Bom* genes.

### The 55C *Bom* cluster is specifically required for the Toll-mediated immune defense

**Fig 2:**
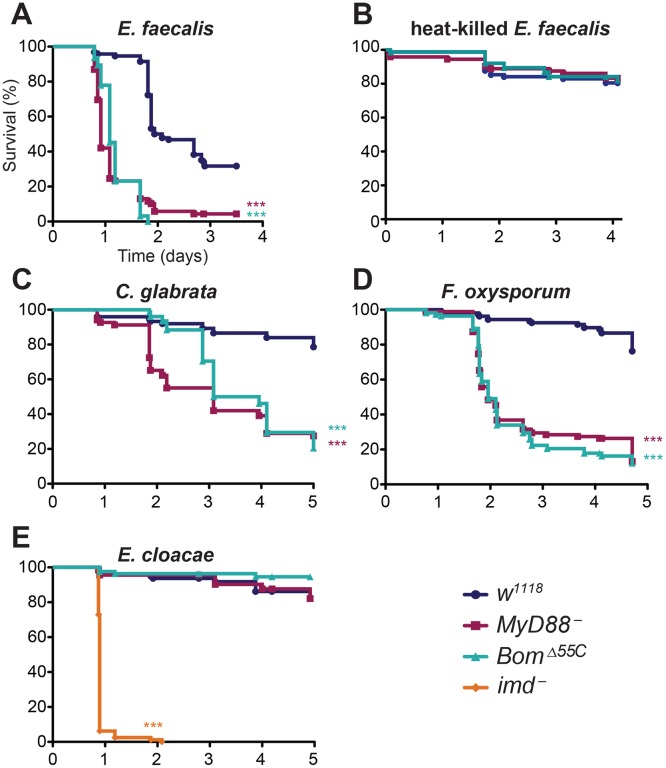
The 55C *Boms* are essential for Toll-mediated defense. Graphs indicate survival at indicated intervals post-infection with (A) *E*. *faecalis*, (B) heat-killed *E*. *faecalis*, (C) *C*. *glabrata*, (D) *F*. *oxysporum*, and (E) *E*. *cloacae*. Each curve represents the pooled results of at least three independent experiments involving 20 or more flies per genotype. Survival curves were compared using the Gehan-Breslow-Wilcoxon test. Significance is shown relative to the wild type (*w* ^*1118*^) and adjusted for multiple comparisons (*** p<0.0003, n.s. = not significant, p>0.0167).

Because Toll signaling induces *Bom* expression, we challenged *Bom* ^*Δ55C*^ adults with *Enterococcus faecalis*, a bacterium that has Lys-type peptidoglycan and therefore specifically induces the Toll pathway. Using septic wounding, we systemically infected adult flies and then monitored survival. In control experiments, we found that flies lacking a functional Toll pathway (*MyD88* ^-^) were much more susceptible to *E*. *faecalis* infection than were flies with wild-type immune competence (*w* ^*1118*^), as reported previously [7, 33]. Following infection, more than 50% of *MyD88* ^-^ flies died within one day and nearly all (>90%) were dead within two days (Fig 2A). In contrast, more than 95% of wild-type adults were alive one day post-infection and more than 50% survived two days or longer.

Strikingly, *Bom* ^*Δ55C*^ flies were as susceptible to *E*. *faecalis* infection as *MyD88* ^-^ flies. Indeed, the survival curves of *Bom* ^*Δ55C*^ and *MyD88* ^-^ flies were almost indistinguishable, suggesting that loss of the 55C *Bom* cluster is as detrimental to defense against this bacterial pathogen as is loss of Toll signaling entirely.

Having observed that *Bom* ^*Δ55C*^ flies rapidly succumb to septic wounding with *E*. *faecalis* (see Fig 2A), we wondered if this phenotype reflected a defective response to wounding or stress rather than infection *per se*. To test this idea, we wounded wild-type and mutant flies with a clean needle or with one dipped in a suspension of heat-killed *E*. *faecalis*. The survival of *Bom* ^*Δ55C*^ flies was markedly better for either challenge compared to septic wounding over the same time period. Specifically, upon either clean wounding or wounding with heat-killed bacteria, more than 75% of *Bom* ^*Δ55C*^ flies survived for four or more days, comparable to the wild type (Fig 2B and S1 Fig). We conclude that active infection, rather than wounding itself or response to PAMP recognition, causes the rapid death of *Bom* ^*Δ55C*^ flies challenged with live *E*. *faecalis*.

Toll mediates resistance not only to a number of bacteria, but also to fungi, including yeast [6, 34]. To determine if this Toll activity is also *Bom*-dependent, we assayed the effect of deleting the 55C *Bom* genes on survival after infection with the yeast *Candida glabrata*. Wild-type flies exhibit significant resistance to *C*. *glabrata*, with over 80% of wild-type flies surviving five days after infection (Fig 2C). In contrast, 50% of *MyD88* ^-^ flies succumbed just two days after being infected. *Bom* ^*Δ55C*^ flies were similarly affected. Although it took *Bom* ^*Δ55C*^ flies slightly longer than *MyD88* ^-^ flies to drop to 50% survival (three days), survival rates were nearly coincident at later time points.

We next tested the survival of *Bom* ^*Δ55C*^ flies after infection with a filamentous fungus, *Fusarium oxysporum*, that also triggers a Toll-dependent immune response. Among wild-type flies, roughly 80% survived for four or more days (Fig 2D). In contrast, both *MyD88* ^-^ and *Bom* ^*Δ55C*^ flies succumbed much more quickly. Specifically, *Bom* ^*Δ55C*^ flies had a median survival of just over two days post-infection, nearly identical to *MyD88* ^-^ flies. We conclude that the 55C *Boms* are also essential for Toll-mediated defense against both a unicellular and a filamentous fungus.

Because the Imd pathway does not appear to regulate *Bom* expression [24], we predicted that the *Bom* genes would be dispensable for Imd-mediated defenses. We could test this hypothesis with *Enterobacter cloacae*, a bacterium that has a DAP-type peptidoglycan and therefore triggers Imd signaling. We used *E*. *cloacae* to infect *Bom* ^*Δ55C*^ flies, as well as control flies lacking *imd* function. Whereas more than 90% of *imd* ^-^ flies died within 24 hours of septic wounding with *E*. *cloacae*, greater than 80% of *Bom* ^*Δ55C*^, *MyD88* ^-^, and wild-type flies survived for four or more days (Fig 2E). Taken together, these studies indicate that the 55C Bom peptides are specifically required in the Toll-mediated, acute phase defense against systemic infection.

### Bom peptides are not required to maintain, protect, or amplify Toll signaling

**Fig 3:**
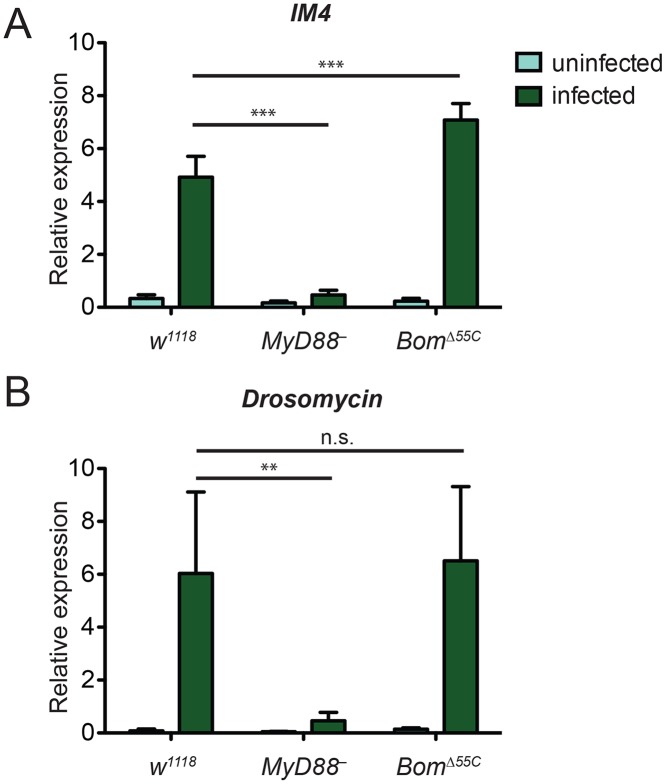
Toll-mediated activation of immune genes is normal in *Bom* ^*Δ55C*^ flies. Transcript levels of the Toll-responsive genes (A) *IM4* and (B) *Drs* in flies were measured in the absence of infection and six hours after infection with *E*. *faecalis*. Expression was measured by qRT-PCR and normalized to that of the ribosomal protein gene *rp49* (*rp49* = 1). Error bars represent SEM. Significance was measured by two-way ANOVA (** p<0.01, *** p<0.001, n.s. = not significant, p>0.05).

Given the similarity in phenotypes between *Bom* ^*Δ55C*^ and *MyD88* ^-^ flies, we wondered if loss of the 55C Boms disrupts Toll signaling. These Boms might, for example, be required to counteract pathogen virulence factors that target Toll signaling. They might also provide positive feedback, spreading and amplifying Toll signaling after initial pathogen detection. According to such models, induction of Toll-responsive genes should be reduced in *Bom* ^*Δ55C*^ flies relative to the wild type. To test this idea, we infected flies with *E*. *faecalis* and used qRT-PCR to measure induction of marker loci. For this purpose, we chose two genes that are strongly expressed upon Toll activation but that lie outside of the 55C cluster: *IM4* and *Drosomycin* (*Drs*). Six hours after *E*. *faecalis* infection, we detected robust expression of *IM4* and *Drs* in the wild type but, as expected, negligible induction in *MyD88* ^-^ (Fig 3A and 3B). In *Bom* ^*Δ55C*^ flies, induction of both Toll-responsive genes was comparable to that in the wild type. In fact, expression of *IM4* was greater in *Bom* ^*Δ55C*^ flies than wild-type flies, perhaps reflecting the enhanced induction of Toll by an unchecked infection.

These experiments reveal that the susceptibility of *Bom* ^*Δ55C*^ flies to microbial infection does not reflect a general block in Toll signaling. Further, they strongly suggest that flies lacking *Bom* gene function have increased susceptibility to *E*. *faecalis*, *C*. *glabrata*, and *F*. *oxysporum* despite normal, Toll-mediated induction of AMP genes.

### Bom peptides mediate infection resistance, not tolerance

Infection resistance is defined as the ability to clear microbes, while infection tolerance is the ability to endure the presence of microbes [35]. Expression of Toll-responsive genes appears unaffected in *Bom* ^*Δ55C*^ flies. Is it the case that AMPs and other Toll-induced effectors kill pathogens in *Bom* ^*Δ55C*^ flies, but the flies nevertheless die due to an inability to tolerate the infection? Alternatively, do *Bom* ^*Δ55C*^ flies succumb because Bom peptides are in fact required to control and clear infections? We set out to distinguish between these hypotheses.

**Fig 4:**
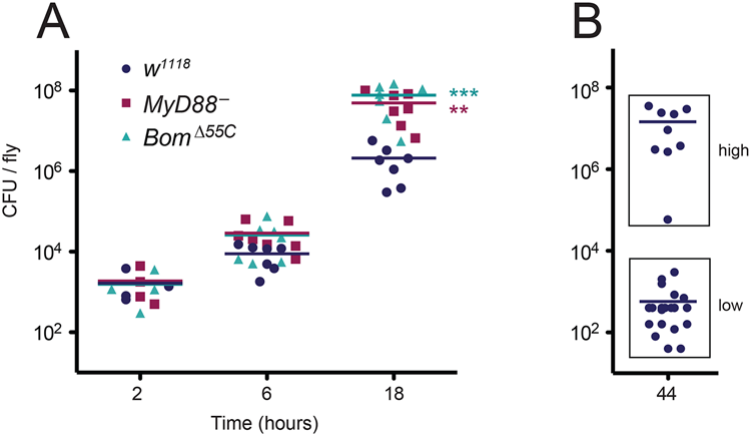
Deletion of the 55C *Bom* genes impairs resistance to *E*. *faecalis* infection rather than tolerance. (A) Points indicate the mean CFU/fly from individual experiments using pools of 5–10 flies per genotype after *E*. *faecalis* infection at the indicated time point. Horizontal bars represent the means of the (four or more) independent experiments shown. Significance was measured by two-way ANOVA and is relative to the wild type (*w* ^*1118*^) at the same time point (** p<0.01, *** p<0.001). (B) CFU of individual wild-type (*w* ^*1118*^) flies at 44 hours post-infection. Horizontal bars represent means. “Low” (<3800 CFU/fly) and “high” (>3800 CFU/fly) populations were measured simultaneously during four independent collections of individual flies. Data were binned (indicated by boxes), and means were calculated separately.

To assess resistance and tolerance, we assayed bacterial load over the course of an *E*. *faecalis* infection, using wild-type, *MyD88* ^-^, and *Bom* ^*Δ55C*^ flies in parallel. Because *Bom* ^*Δ55C*^ and *MyD88* ^-^ flies have a median survival after *E*. *faecalis* infection of about 23 hours, time points were taken at intervals up to 18 hours. At two hours post-infection, all flies had similar bacterial loads (Fig 4A). At later time points, however, differences emerged. At six hours, the bacterial load was on average 3-fold greater in *Bom* ^*Δ55C*^ than in the wild type. The bacterial load of *MyD88* ^-^ flies was similarly elevated relative to wild-type flies. At 18 hours, both *Bom* ^*Δ55C*^ and *MyD88* ^-^ flies had a bacterial load at least 20-fold greater than did wild-type flies. This elevation in bacterial load in *MyD88* ^-^ and *Bom* ^*Δ55C*^ flies over the course of infection suggests that Toll signaling in general, and Bom peptides specifically, contribute to resistance.

To further explore the questions of resistance and tolerance, we measured bacterial load in wild-type flies at 44 hours post-infection, an interval slightly shorter than their median survival time (48 hours). If Boms contribute to resistance rather than tolerance, the bacterial load in wild-type flies at 44 hours post-infection should be similar to the bacterial load of *Bom* ^*Δ55C*^ and *MyD88* ^-^ flies at 18 hours post-infection.

The response of the wild type to *E*. *faecalis* infection necessitated a minor modification in our protocol for assaying bacterial load. In particular, some wild-type flies appear to clear *E*. *faecalis* infection, as evident in survival curves that do not reach 0% survival, but instead level out at an intermediate value (see, for example, Fig 2A). Foreseeing a bimodal distribution of bacterial loads among wild-type flies at 44 hours—some clearing infection and others not—we measured bacterial load for this time point in individual flies, rather than in groups. Measured in this way, there were indeed two groups with quite distinct bacterial loads. Of 32 wild-type flies still alive at this time point, 23 had a bacterial load less than 4,000 colony forming units (CFU) (Fig 4B, “low”). These low CFU flies presumably represent the fraction of the population that survives infection. The other nine wild-type flies had bacterial loads at 44 hours ranging from 60,000 to 36,000,000 CFU, comparable to those of *Bom* ^*Δ55C*^ and *MyD88* ^-^ flies at 18 hours (compare Fig 4B, “high” to Fig 4A, 18h). Thus wild-type flies that succumb to infection do so at a bacterial load comparable to that in the mutants. We conclude that the *Bom* ^*Δ55C*^ flies succumb to infection more quickly than the wild type due to a defect in resistance.

### Deleting a subset of 55C *Bom* genes reveals overlap in *Bom* gene activity

Our experiments with *Bom* ^*Δ55C*^ flies demonstrate that the 55C *Bom* cluster is required to provide Toll-mediated resistance to our test set of pathogens. Is the entire gene cluster required? If not, to what extent do the 55C genes overlap in function? To address these questions, we set out to assay how flies expressing a subset of the 55C *Bom* genes fare when infected with the same test set.

In the course of investigating the 55C cluster, we came across a publically available stock carrying an insertion in the 3’ UTR of *IM2* of a *MiMIC* (Minos-mediated integration cassette) transposon [36]. By inducing the excision of this *MiMIC* element, we obtained two chromosomes that had lost the insertion. In one case the excision was imprecise. The resulting chromosome, hereafter *Bom* ^*Δleft*^, lacks *IM2* and the five *Bom* genes to the left (proximal) of *IM2*, but retains the four *Bom* genes to the right (distal) of *IM2* (see Fig 1C). The other chromosome, hereafter *IM2* ^*ΔMi*^, had undergone a precise excision and thus provided a valuable control for subsequent studies.

**Fig 5:**
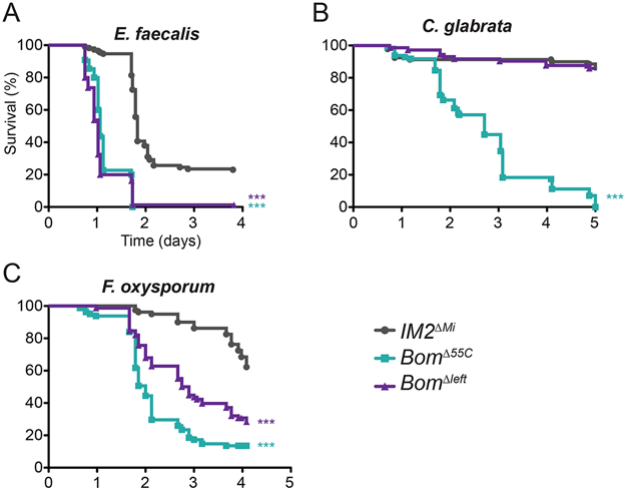
Loss of a subset of the 55C *Bom* genes has an intermediate effect on survival. Graphs indicate survival at indicated intervals post-infection with (A) *E*. *faecalis*, (B) *C*. *glabrata*, and (C) *F*. *oxysporum*. Each curve represents the pooled results of at least three independent experiments involving 20 or more flies per genotype. Survival curves were compared using the Gehan-Breslow-Wilcoxon test. Significance is relative to *IM2* ^*ΔMi*^ and adjusted for multiple comparisons (*** p<0.0003, n.s. = not significant, p>0.0167).

To assay the activity of the four 55C *Bom* genes present in the imprecise excisant, we challenged *Bom* ^*Δleft*^, *IM2* ^*ΔMi*^, and *Bom* ^*Δ55C*^ flies by infection and monitored survival. For all pathogens tested, we defined the phenotype of *Bom* ^*Δ55C*^ flies as lacking resistance and that of *IM2* ^*ΔMi*^ flies as having full resistance over a four to five day post-infection interval. Based on this scale, *Bom* ^*Δleft*^ flies lacked resistance to *E*. *faecalis*, exhibited partial resistance to *F*. *oxysporum*, and had full resistance to *C*. *glabrata* (Fig 5A–5C). The *Bom* ^*Δleft*^ chromosome thus provided a subset of the 55C *Bom* cluster immune activity.

We draw three conclusions from the experiments shown in Fig 5. First, the wild-type resistance of *Bom* ^*Δleft*^ flies to challenge with *C*. *glabrata* demonstrates that the complete 55C *Bom* gene set is not a prerequisite for *Bom* function. Second, the fact that *Bom* ^*Δleft*^ flies have partial resistance to *F*. *oxysporum* indicates that at least some *Bom* genes overlap in specificity. Third, resistance to some pathogens requires more than one Bom peptide. In particular, wild-type resistance to *F*. *oxysporum* must require at least one of the genes present in *Bom* ^*Δleft*^ but deleted in *Bom* ^*Δ55C*^, as well as one or more of the genes deleted in in *Bom* ^*Δleft*^. In and of themselves, these studies do not reveal whether Bom peptides have a narrow- or broad-spectrum of activity, but do provide clues in this regard, as addressed in the discussion.

## Discussion

### A gene family essential for infection resistance

We report here that Toll-mediated defenses against a bacterium, yeast, or filamentous fungus require *Bom* gene function. Having reached this conclusion based on loss-of-function phenotypes, we note that such an approach has only rarely been applied to the role of innate immune effectors [37–40]. The paucity of such studies has several likely causes. First, many effector genes, such as the *Boms* and the known AMP genes, encode peptides that are sufficiently small as to be relatively refractory to random mutagenesis. Second, large-scale screens that rely on reporter genes are useful for identifying lesions that block pathogen recognition or response pathway signaling, but opaque to disruptions in more downstream processes.

Perhaps the biggest obstacle, real or imagined, to loss-of-function studies of immune effectors has been the existence of families of closely related genes. One might reasonably expect significant overlap in gene function, meaning that multiple family members would need to be inactivated to uncover reliable phenotypes. Instead, researchers interested in knockout phenotypes have typically focused on those examples where paralogs are absent. Thus, for example, the loss-of-function study demonstrating that disruption of a mouse *cathelicidin* gene promoted invasive skin infection with Group A *Streptococcus* [40] relied on the fact that mice, unlike some other mammals, encode only one member of this gene family.

### Bomanin peptide function

How do Bom peptides promote infection resistance? One possibility is that the Bom peptides support cellular immune function. To explore this question, we infected *Bom* ^*Δ55C*^ flies with *Staphylococcus aureus*. Defense against *S*. *aureus* has been shown to involve cellular immune activities to a greater extent than for some other Toll-activating bacteria, including *E*. *faecalis* [41–46]. Although *Bom* ^*Δ55C*^ flies succumbed to *S*. *aureus* infection more quickly than did the wild type, *Bom* ^*Δ55C*^ survival was indistinguishable from that of *MyD88* ^-^ and the genetic background control, *IM2* ^*ΔMi*^ (S2A Fig). We thus found no evidence that the 55C *Bom* gene cluster contributes to Toll-independent cellular mechanisms of resistance. In additional studies of cellular immune functions, we found neither defects in wound site melanization in *Bom* ^*Δ55C*^ adults nor any deficiency in hemocyte number in *Bom* ^*Δ55C*^ larvae (S2B and S2C Fig). These experiments do not, however, preclude a role for for the Boms in Toll-dependent cellular immunity.

A likely alternative is that the Bom genes encode antimicrobial peptides (AMPs). Like many AMPs, Bom peptides are short, secreted, have intramolecular disulfide bonds, and undergo post-translational processing. Although short-form Bom peptides would be the shortest characterized *Drosophila* AMP, the mature form of Drosocin is just three amino acids longer [47]. Furthermore, both the Boms and the known AMPs populate the upper echelons of the sets of genes most highly upregulated upon activation of the Toll pathway. Specifically, at 12, 24, and 96 hours after natural infection by the fungus *Beauveria bassiana*, the 30 most highly upregulated genes include five or more *Bom* family members and five or more known AMP genes [24]. Additionally, mass spectrometry data indicate that a number of Bom peptides are as abundant as known AMPs, which after infection reach concentrations of 10–100 μM in the hemolymph [23, 32].

The structure and sequence of the 55C *Bom* cluster suggests that the *Bom* family arose by multiple gene duplications. Such events are enriched among loci involved in pathogen resistance and provide the opportunity for divergence in gene function driven by positive selection [48, 49]. One example is the *Peptidoglycan Recognition Protein* (*PGRP*) gene family, which consists of 13 genes, some clustered, encoding 19 proteins. While all PGRPs share a peptidoglycan-recognition domain, the functions of the proteins vary considerably [50]. Some activate the Toll or Imd signaling pathways and promote phagocytosis, autophagy, and melanization. Others suppress the Imd pathway, protecting commensal gut bacteria. A third class has direct bactericidal activity.

Gene duplications need not, however, result in functional divergence. Rather, there are circumstances in which gene duplications instead lead to changes in the level, location, or timing of expression of what is essentially the same gene product. Minor sequence variation will arise, but in general the coding regions will not bear the hallmarks of positive selection [51–53].

Comparing the effects of eliminating some or all *Bom* genes in the Bom 55C cluster, we observe differential effects on resistance to particular pathogens. How does this observation fit with the alternative potential outcomes for gene duplication?

If functional divergence has occurred, we would expect that at least some Bom peptides have a narrow-spectrum effector activity, i.e., are specific for a particular pathogen or set of pathogens. One or more of the six *Bom* genes deleted in the *Bom* ^*Δleft*^ chromosome would be specific for *E*. *faecalis*, while one or more of the four 55C *Bom* genes remaining in *Bom* ^*Δleft*^ would protect specifically against *C*. *glabrata*. There would need to be at least two peptides specific for *F*. *oxysporum*: one or more of the six genes deleted in the *Bom* ^*Δleft*^ chromosome, as well as one or more of the four remaining 55C *Bom* genes.

Although our data can accommodate a narrow-spectrum activity model, we favor the idea that Bom peptides have a common, broad-spectrum activity. In this scenario, resistance to different pathogens would require different total Bom peptide levels, as could be produced by variation in the numbers of *Bom* genes. In particular, defense against *E*. *faecalis* would require the greatest level of Bom activity, while *C*. *glabrata* would require the least. Defense against *F*. *oxysporum* would require a level intermediate to that required for *E*. *faecalis* and *C*. *glabrata*.

When considered *in toto*, our survival data support this broad-spectrum effector model: the predicted hierarchy of Bom activity levels required for particular pathogens parallels the overall virulence levels for these pathogens (*E*. *faecalis* > *F*. *oxysporum* > *C*. *glabrata*). This holds true when virulence is measured either by the proportion of wild-type flies that succumb to infection or by the rates at which the mutants succumb after infection (compare Fig 2A, 2C and 2D). The parallel between required Bom activity level and pathogen virulence makes sense if we make the reasonable assumption that the broad-spectrum activity of Bom peptides is more efficient and rapid at higher concentrations.

Future comprehensive consideration of the Bom genes will necessitate taking into account four additional loci. Two of these, *CG5778* and *CG5791*, are *Bom* genes located outside the 55C cluster. The remaining two, *IM4* and *IM14*, encode peptides that lack a CXXC motif, but nevertheless exhibit sequence similarity with Bom family members. Furthermore, the IM4 and IM14 peptides, like the Bomanins listed in Fig 1A, are small, secreted, specific to the *Drosophila* genus, and robustly induced by Toll.

### The functional relationship of Bom peptides and Drosomycin

Lemaitre and colleagues have reported that overexpression of a UAS-*Drs* construct using a ubiquitous GAL4 driver restores *F*. *oxysporum* resistance to flies lacking both Toll and Imd pathway function [54]. In our studies, however, we found that *Bom* ^*Δ55C*^ flies induce *Drs* expression upon infection (see Fig 3B), but succumb as rapidly as flies lacking Toll signaling (see Fig 2). Why was a requirement for *Bom* gene function not apparent in the Lemaitre study? One possibility is that loading the flies with high levels of Drosomycin prior to infection obviates the need for additional Toll-induced loci, including the *Bom* genes. An alternative explanation lies in the fact only inducible *Bom* expression was blocked in the Lemaitre study, whereas our study eliminated all *Bom* gene function. It might be that the synergistic activity of both Drosomycin and Boms is required to defend against *F*. *oxysporum*, but that a basal, Toll-independent level of *Bom* expression is sufficient for this synergy. In support of this idea, RNA-seq data from modENCODE demonstrate that expression of many *Bom* genes is robust even in the absence of infection [55].

### Contribution of Bom activity to Toll-mediated defense

Given that innate immune signaling pathways direct expression of large batteries of effector genes upon infection, including many AMPs, one might have expected that disabling a small subset of that repertoire would have only minor effects on the overall immune response. That is not what we observe. Instead, elimination of Bom activity is indistinguishable in phenotype from loss of the entire Toll-mediated immune defense for the pathogens tested, although Toll signaling is intact. We envision at least four explanations for the essential role of the Bom gene family:

1. The Boms have a unique and central role in Toll-mediated defense. This might seem unlikely, given that the Bom family is apparently specific to the *Drosophila* genus and thus represents a relatively young family of effectors. However, such a model is, in fact, in keeping with recent studies on genes that are essential in the sense of being required for viability. In particular, we now know that essentiality is found in equal proportions among old and young *Drosophila* genes, where age is measured on the scale of divergence time between species [56]. It could therefore be that a gene family and associated function that arose fairly recently has become a dominant and essential feature of the innate immune response.
2. The Boms are essential for Toll-mediated responses specific to certain microbial pathogens. We know from a variety of expression studies that the entire Toll effector repertoire is upregulated regardless of the source of the activating signal, PAMP or otherwise. By this model, Toll activates a large number of effectors, each attacking a subset of pathogens, rather than collectively fighting a common target pathogen. If so, there should be additional pathogens for which Toll is required and the Bom peptides are not.
3. The Bom genes and other effectors synergize, such that loss of just a single factor disrupts defense as strongly as loss of all components. By this model, innate immunity involves a network of effector functions that comprise multiple hubs, each making a vital contribution to defense.
4. The *Bom* family and other components of the Toll repertoire are each expressed at the minimal level required for resistance. There is good evidence that immune activation requires an energy tradeoff with metabolic processes [57, 58]. As a result, limiting the resources used by an immune response can be beneficial to overall health.

By either of the last two models, knocking out other effector families should result in the same phenotype observed with the *Bom* ^*Δ55C*^ deletion, i.e., inactivation of Toll defenses. Testing this prediction thus holds promise for a broader understanding of innate immune effector function *in vivo*.

## Materials and Methods

### Flies and mutant generation

Flies were raised at 25°C on standard cornmeal agar media. The *w* ^*1118*^ strain was used as the wild type. *MyD88* ^-^ flies were *MyD88* ^*kra1*^, and *imd* ^-^ flies were *imd* ^*shadok*^. All flies were homozygous for the listed mutations.

TALEN mutagenesis was conducted as previously described [59]. *In vitro* transcription was conducted using the Ambion Megascript kit with a Promega 5’-cap analog. TALEN transcripts were injected into a fly line containing a *MiMIC* element in the *IM2* gene (*y* ^*1*^ w*; *Mi[MIC]IM2* ^*MI01019*^, Bloomington stock center, #32727). The *MiMIC* element carries the mini-*yellow* marker. F0 flies were crossed to *yw*; *Sco/CyO* and the resulting *y* ^-^ F1 flies were collected and crossed to *yw*; *Sco/CyO*. Stocks were established and genotyped using Phusion polymerase and primers flanking the predicted deletion end points. We confirmed the exact endpoints of the deletions by sequencing the PCR product.

Excision of the *MiMIC* element from *Mi[MIC]IM2* ^*MI01019*^ was conducted as described previously [60] with the transposase source coming from stock *y* ^*1*^ w*; *sna* ^*Sco*^ */SM6a*, *P{hsILMiT}2*.*4* (obtained from Bloomington stock center, stock #36311).

### Microbial culture

Microbes were prepared for infection experiments as follows. *Enterococcus faecalis* strain NCTC 775 (ATCC 19433) and *Enterobacter cloacae* were cultured in LB media at 37°C and concentrated to OD_600_ = 10 in 20% glycerol. For heat-killed *E*. *faecalis* challenges, cultures were concentrated to OD_600_ = 10 in 20% glycerol and then boiled for 30 minutes. *Staphylococcus aureus* subsp. *aureus* Rosenbach (ATCC 29213) was cultured in LB media at 37°C and concentrated to OD_600_ = 0.5 in 20% glycerol. *Candida glabrata* strain *CBS 138* (ATCC 2001) was cultured in YPD media at 30°C and concentrated to OD_600_ = 50 in 20% glycerol for infection. *Fusarium oxysporum* f. sp. *lycopersici* (obtained from the Fungal Genetics Stock Center) was cultured on oatmeal agar plates for 7–10 days at 29°C before being strained through steel wool to isolate spores. Purified spores were resuspended in 20% glycerol and stored at -80°C until infection.

### *Drosophila* infection, survival analysis, and bacterial load analysis

For infection, at least 20 2–7 day old, male flies per genotype were anaesthetized and septically wounded in the anterior lateral thorax with a size 000 insect pin dipped in a suspension of pathogen. Survival analysis was conducted essentially as described previously [61]. After infection, flies were incubated at 25°C (live or heat-killed *E*. *faecalis* or live *S*. *aureus*) or 29°C (clean wounding, *F*. *oxysporum*, *C*. *glabrata*, and *E*. *cloacae*), and the number of dead flies were counted at least once per day for the given time interval. Flies that died within 6 hours of infection were excluded from analysis, except in challenges with a clean needle or heat-killed *E*. *faecalis*. Colony forming units (CFUs) were assayed as described previously [62].

### Cellular immunity assays

For the wound site melanization assay, 2–7 day old males were wounded as described above with a clean insect pin, incubated for three days at 25°C, and visually inspected for melanization at the wound site. Hemocytes from five wandering third instar larvae per genotype per experiment were obtained and counted as previously described [63].

### Gene expression quantitation

RNA was prepared using Trizol (Ambion) from 2–7 day old males, and first-strand cDNA was synthesized with the SuperScript II kit (Invitrogen). Quantitative RT-PCR was performed on an iQ5 cycler (BioRad) using iQ SYBR Green Supermix (BioRad).

### Data analysis

GraphPad Prism was used to run statistical analyses. Survival data were plotted on a Kaplan-Meier curve and the Gehan-Breslow-Wilcoxon test was used to determine significance. The Gehan-Breslow-Wilcoxon test is recommended for analysis of infection with sublethal pathogen doses; where a sublethal dose is defined as a dose where some proportion of wild-type flies survive. Hemocyte count data were analyzed by one-way ANOVA using the Bonferroni post method. Quantitative RT-PCR and bacterial load data were analyzed by two-way ANOVA using the Bonferroni post method.

## Supporting Information

S1 Table Mature Bom peptide sequences. Comparison of mature sequences for the three classes of Bom peptides. Bom motif sequences (as shown in Fig 1A) are highlighted in red. An asterisk indicates that processing was confirmed by published mass spectrometry [23, 25]. Those studies indicate that a number of Bom peptides (indicated by dagger) undergo C-terminal amidation.
(PDF)

S1 Fig Deletion of 55C *Bom* gene cluster does not affect ability to recover from wounding alone. Survival rate of flies after wounding with a clean needle. Each curve represents the pooled results of three independent experiments involving 20 or more flies per genotype. Experiment-wide Log-rank test shows no significant difference between curves for any genotype.
(TIF)

S2 Fig The 55C *Bom* genes do not appear to contribute to cellular immunity. (A) Survival at indicated intervals post-infection with *S*. *aureus*. Each curve represents the pooled results of three independent experiments involving 20 or more flies per genotype. Survival curves were compared using the Gehan-Breslow-Wilcoxon test. Significance is relative to *w* ^*1118*^ and adjusted for multiple comparisons (*** p<0.00017, n.s. = not significant, p>0.0083). There is no significant difference between *MyD88* ^-^, *Bom* ^*Δ55C-*^, and *IM2* ^*ΔMi*^. (B) Proportion of flies to develop melanization at wound site three days after wounding with a clean needle. (C) Hemocyte counts in uninfected larvae of the indicated genotypes. Six groups of five larvae per genotype were counted and averaged. Error bars represent SEM. Significance was measured by one-way ANOVA (** p<0.01, n.s. = not significant, p>0.05).
(TIF)
