# Supplementary material for: An Effector Peptide Family Required for Drosophila Toll-Mediated Immunity
Source: PLoS Pathog. 2015 Apr 27;11(4):e1004876. doi: 10.1371/journal.ppat.1004876 (PMC4411088; doi:10.1371/journal.ppat.1004876)
Supplement: S1 Table — Comparison of mature sequences for the three classes of Bom peptides. Bom motif sequences (as shown in Fig 1A) are highlighted in red. An asterisk indicates that processing was confirmed by published mass spectrometry [23, 25]. Those studies indicate that a number of Bom peptides (indicated by dagger) undergo C-terminal amidation. (PDF) [file ppat.1004876.s001.pdf]

Gene Name	Actual (*) or Predicted Mature Peptide Sequence
**Short-Form Boms**	
IM1 *†	GNVIINGDCRVCNVHG
CG18107	GNVIINGDCVNCNVRG
IM2 *†	GNVVINGDCKYCNVHG
IM3 *	GNVIINGDCRVCNVRA
CG15065	GNVIINGDCRHCNVRG
CG15068	GNVIINGDCKVCNIRGD
**Tailed Boms**	
CG43202	GDIIVHGNCNDCTARATKNSAHLSIKFTRRW
CG16836	GQVYINGKCIDCNKPDNDPGIIIPPDHKSAGSMSYTLTSGAIFFGIIYH<br>IFS
CG5778	AAVYIGGGCYDCNPPGGQGPGVYTGGNGGRGGGGGYNAGGGGGGYYNGG<br>GGGGGGRRPVYSGNFGPGYGNGGGGGGGGYGGGGGGGYDDGGLTQIISG
**Bicipital Boms**	
IM23 *	GNVIIGGVCQDCSPPVAENVVVGGQSYRTGRPGQGTVYINSPGAYLGAL<br>DGPIRRTGAGGGGGGGAQYPDGYSGRLPGGTYLHNKDCVGCSISGGGD
CG15067	GKVTINGKCVNCSHDQTTTTTHKPTSGKGSGGRTTARPSSRSSPARGRP<br>SWDDDDDDDLTGDWALHQSAGGTQYIGRRSKRQSRGGQYIDLGGSGGRG<br>GGGGWAGSGITTIDSSGYPGGTLVRNSDCVGCNIRG
CG5791	STVVVNGVCLTCPNPNGEPVYLDGQQYRSFSSSPGDGNVVISRGNDGSG<br>GGGGTIYRRGGNTIVNGRCQHCNVDPY
