# Supplementary figures and images for: An Effector Peptide Family Required for Drosophila Toll-Mediated Immunity
Source: PLoS Pathog. 2015 Apr 27;11(4):e1004876. doi: 10.1371/journal.ppat.1004876 (PMC4411088; doi:10.1371/journal.ppat.1004876)

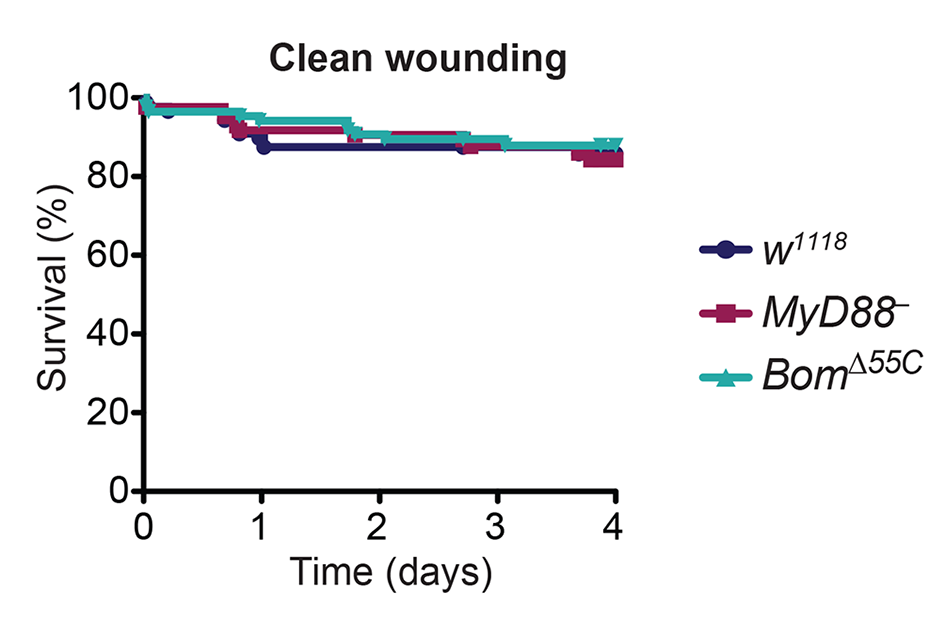

Supplement: S1 Fig — Survival rate of flies after wounding with a clean needle. Each curve represents the pooled results of three independent experiments involving 20 or more flies per genotype. Experiment-wide Log-rank test shows no significant difference between curves for any genotype. (TIF) [file ppat.1004876.s002.tif]

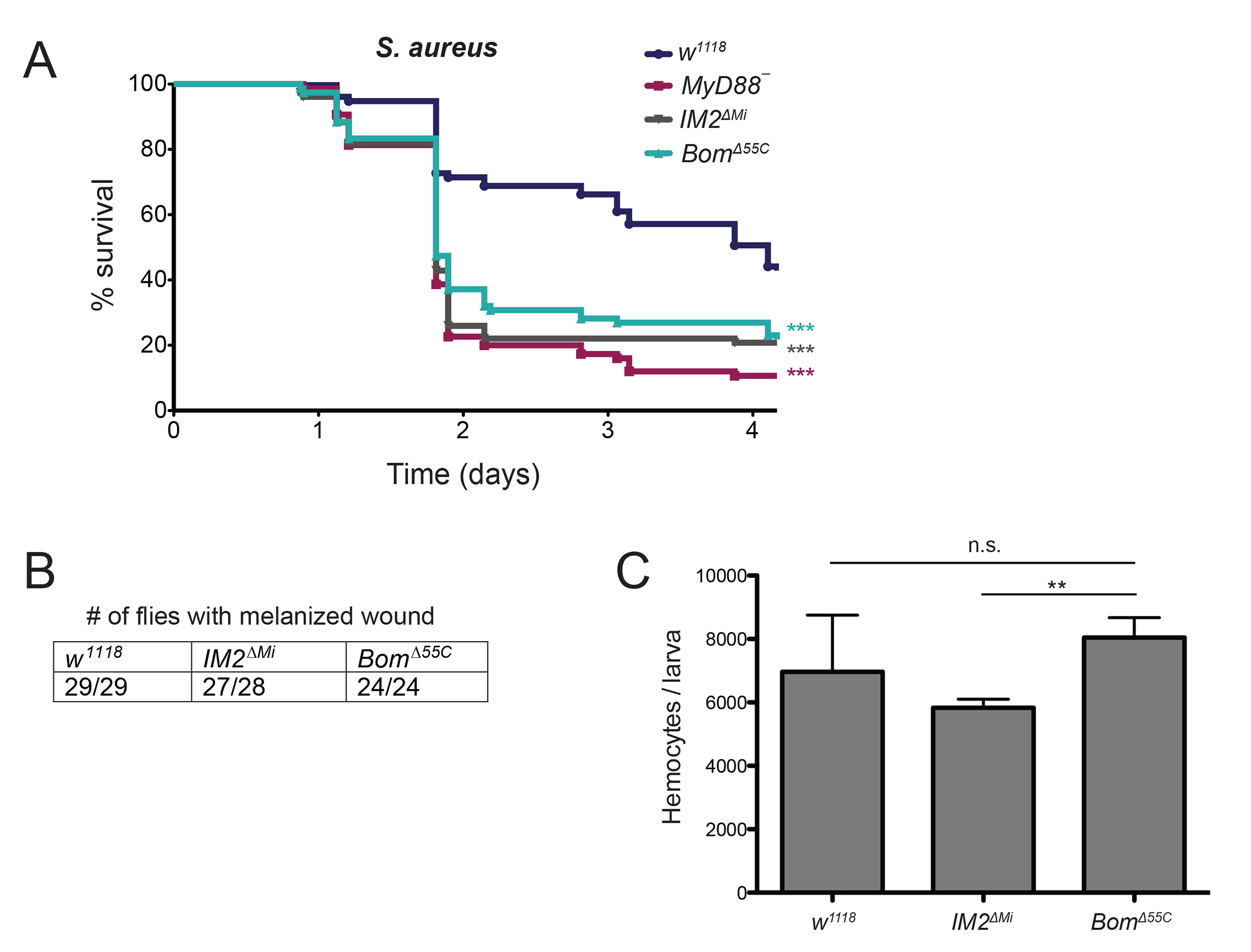

Supplement: S2 Fig — (A) Survival at indicated intervals post-infection with S. aureus. Each curve represents the pooled results of three independent experiments involving 20 or more flies per genotype. Survival curves were compared using the Gehan-Breslow-Wilcoxon test. Significance is relative to w 1118 and adjusted for multiple comparisons (*** p<0.00017, n.s. = not significant, p>0.0083). There is no significant difference between MyD88 -, Bom Δ55C-, and IM2 ΔMi. (B) Proportion of flies to develop melanization at wound site three days after wounding with a clean needle. (C) Hemocyte counts in uninfected larvae of the indicated genotypes. Six groups of five larvae per genotype were counted and averaged. Error bars represent SEM. Significance was measured by one-way ANOVA (** p<0.01, n.s. = not significant, p>0.05). (TIF) [file ppat.1004876.s003.tif]
